# Human Nasal Inferior Turbinate-Derived Neural Stem Cells Improve the Niche of Substantia Nigra Par Compacta in a Parkinson’s Disease Model by Modulating Hippo Signaling

**DOI:** 10.1007/s13770-024-00635-3

**Published:** 2024-04-10

**Authors:** Junwon Choi, Sun Wha Park, Hyunji Lee, Do Hyun Kim, Sung Won Kim

**Affiliations:** 1 Department of Otolaryngology-Head and Neck Surgery, College of Medicine, Seoul St. Mary’s Hospital, The Catholic University of Korea, Seoul, Republic of Korea; 2 Postech-Catholic Biomedical Engineering Institute, College of Medicine, The Catholic University of Korea, Seoul, Republic of Korea

**Keywords:** Cell-based therapy, Regenerative medicine, Parkinson’s disease, Human nasal turbinate derived stem cells, Niche control

## Abstract

**Background:**

Parkinson’s disease (PD) is one of the most prevalent neurodegenerative diseases, following Alzheimer’s disease. The onset of PD is characterized by the loss of dopaminergic neurons in the substantia nigra. Stem cell therapy has great potential for the treatment of neurodegenerative diseases, and human nasal turbinate-derived stem cells (hNTSCs) have been found to share some characteristics with mesenchymal stem cells. Although the Hippo signaling pathway was originally thought to regulate cell size in organs, recent studies have shown that it can also control inflammation in neural cells.

**Methods:**

Dopaminergic neuron-like cells were differentiated from SH-SY5Y cells (DA-Like cells) and treated with 1-Methyl-4-phenylpyridinium iodide to stimulate Reactive oxidative species (ROS) production. A transwell assay was conducted to validate the effect of hNTSCs on the Hippo pathway. We generated an MPTP-induced PD mouse model and transplanted hNTSCs into the substantia nigra of PD mice via stereotaxic surgery. After five weeks of behavioral testing, the brain samples were validated by immunoblotting and immunostaining to confirm the niche control of hNTSCs.

**Results:**

*In-vitro* experiments showed that hNTSCs significantly increased cell survival and exerted anti-inflammatory effects by controlling ROS-mediated ER stress and hippocampal signaling pathway factors*.* Similarly, the *in-vivo* experiments demonstrated an increase in anti-inflammatory effects and cell survival rate. After transplantation of hNTSCs, the PD mouse model showed improved mobility and relief from PD symptoms.

**Conclusion:**

hNTSCs improved the survival rate of dopaminergic neurons by manipulating the hippocampal pathway through Yes-associated protein (YAP)/transcriptional coactivator with a PDZ-binding motif (TAZ) by reducing inflammatory cytokines. In this study, we found that controlling the niche of hNTSCs had a therapeutic effect on PD lesions.

**Supplementary Information:**

The online version contains supplementary material available at 10.1007/s13770-024-00635-3.

## Introduction

Parkinson’s disease is the second most prevalent neurodegenerative disorder and affects motor function, causes resting tremors, and affects the autonomic nervous system [1, 2]. The etiology of the disease is not clear, but alpha-synuclein (αSyn) deposition and the Lewy-body (LB) formation have been observed to affect the loss of dopaminergic neurons in the substantia nigra par compacta through the post mortem analyses [3, 4]. Syn is a small abnormal protein that is found in various cell types, mostly in the presynaptic terminals of the central nervous system [5]. The domains of αSyn play a role membrane attachment and fibril aggregation. The N-terminal domain of αSyn is composed of an amphipathic α-helix structure which has high affinity to the mitochondrial membrane. The C-terminal domain contains numerous negatively charged amino acids including Ser129. This promotes αSyn aggregation by strengthening the interaction between metal ions and proteins [6–8]. In contrast, ROS are by-products of cellular metabolism in the mitochondria. ROS is necessary for maintaining mitochondrial homeostasis through the mitophagy quality control system [9–11]; however, aggregated αSyn bounds to the mitochondria membrane and disrupts electron transfer system (ETS) complex I, which leads to increase ROS levels in mitochondria [10, 12]. Excessive ROS levels alter mitochondrial membrane permeability and lead to mitochondrial dysfunction and cell death [13, 14]. Thus, αSyn deposition in the central nerve system produces ROS, which disrupts mitochondrial homeostasis [15–18].

The Hippo signaling pathway controls organ size, cancer development, and tissue regeneration [19, 20]. Recent studies have shown that the Hippo-signaling pathway also plays a critical role in controlling neuroinflammation, neuronal cell differentiation, and neuronal cell death [21, 22]. The canonical Hippo-signaling pathway is mainly composed of mammalian Ste20-like kinases 1/2 (MST 1/2), large tumor suppressor 1/2 (LATS 1/2), YAP/TAZ. MST 1/2 phosphorylates LAT 1/2, which leads to the phosphorylation of the downstream YAP/TAZ under oxidative stress. Phosphorylation of YAP/TAZ prevents their translocation into the nucleus and interaction with cytosolic protein 14-3-3, resulting in cell death [23]. In contrast, Unphosphorylated YAP/TAZ translocates to the nucleus and binds to the TEAD family of transcription factors, promoting the expression of genes related to cell proliferation, differentiation, and survival [24, 25].

Mesenchymal stem cells (MSCs) are promising for cell therapy because of their immunomodulatory and therapeutic functions in lesions. A sufficient number of cells are needed for clinical trials. MSCs have various sources, such as bone marrow, umbilical cord blood, and adipocytes, and are relatively easy to obtain. Several studies have investigated the therapeutic effects of MSCs [26, 27], and MSCs have been observed to secrete anti-inflammatory cytokines with immune suppression effects [28, 29]. Human nasal turbinate-derived neural crest stem cells (hNTSCs) represent a new source of light for the treatment of neurodegenerative diseases. hNTSCs show characteristics similar to MSCs, are relatively functional, and differentiate into neurons and astrocytes. As hNTSCs are derived from the neural crest and have neural lineage features, hNTSCs have better compatibility than MSCs [30–32]. Restoring the nigrostriatal pathway through dopaminergic neuron regeneration is a promising method for the treatment of Parkinson’s [33, 34]. Furthermore, improving the survival rate of the transplanted cells is important for successful therapeutic effects [35, 36]. In this study, we generated a Parkinson’s disease model using SH-SY5Y cells that differentiated into dopaminergic neurons and treated them with 1-methyl-4-phenyl pyridinium (MPP^+^) and utilized a MPTP-mediated mice model. The substantia nigra par compacta was relieved by niche control of hNTSCs in the lesion.

## Materials and methods

### hNTSCs generation and cell culture

The study using hNTSCs followed the guidelines of the Institutional Review Board of Seoul St. Mary’s Hospital (KC08TISS0341), Catholic University of Korea, including informed consent regulations and the Declaration of Helsinki. Before surgery, the participants provided written informed consent to participate in the study. The tissue was donated by patient who conducted hypertrophied nasal inferior turbinate volume reduction surgery. hNTSCs were isolated from the discarded tissue following partial turbinectomy using previously described methods [30]. First, the tissues were washed with 0.9% saline and phosphate-buffered saline (Thermo Fisher Scientific). The specimens were cut into 1 mm pieces and placed in a culture dish. Finally, a sterilized glass cover slide was placed on top. The tissue was placed in a humidified incubator and maintained at a temperature of 37 °C with 5% CO_2_. The specimen was cultured in α-minimum essential medium (α-MEM; Thermo Fisher Scientific) which was supplemented with 1% penicillin/streptomycin (Invitrogen, CA, USA), and 10% fetal bovine serum (FBS; Thermo Fisher Scientific). The culture medium was changed every two days for three weeks. The cells were harvested from the tissue by removing the glass cover slide and using 0.25% trypsin and 1 mM EDTA solution. hNTSCs were cultured and expanded for use in experiments.

### hBM-MSC generation and cell culture

Healthy donors provided human bone marrow aspirates from the iliac crest following approval from the Institutional Review Board of Seoul St. Mary’s Hospital (KC10CSSE0651). Bone marrow aspirates were sent to the good manufacturing practice-compliant facility of the Catholic Institute of Cell Therapy (Seoul, Korea, http://www.cic.re.kr) for hBM-MSCs isolation, expansion, and quality control, with written consent from the participants [26]. The hBM-MSCs were cultured in Dulbecco’s modified Eagle’s medium (DMEM) supplemented with 1% penicillin/streptomycin (Invitrogen) and 20% FBS (Thermo Fisher Scientific). The cells were incubated at 37 °C in a humidified atmosphere containing 5% of CO_2_.

### SH-SY5Y cell culture and differentiation

SH-SY5Y cells were purchased from the Korean Cell Line Bank (Seoul, Korea). The cells were cultured in the humidified incubator, which maintained 37 °C and contained 5% CO_2_, in α-MEM supplemented with 1% penicillin/streptomycin (Invitrogen), and 10% FBS (Thermo Fisher Scientific). SH-SY5Y cells were seeded at a density of 3 × 10^4^ cells in a 6-well-plate, and the differentiation medium was changed according to a previously described protocol [37].

### Trans-well assay

DA-like cells (1 × 10^6^ cells of DA-like cells were cultured in the bottom chamber, treated with 500 mM MPP^+^ in culture medium overnight, and the following day, the medium was replaced with fresh medium. One day later, 5 × 10^5^ hNTSCs and hBC-MSCs were cultured in the upper chamber, after being placed at the chamber bottom, and incubated overnight.

### 1-methyl-4-phenyl-1,2,3,6-tetrahydropyridine (MPTP)-induced PD mouse model generation

Seven-week-old C57BL/6N mice were obtained from Orient Bio (Gyeonggi-do, South Korea). Ten mice were randomly assigned to one of four groups: control, MPTP-saline, MPTP_hNTSCs, and MPTP_hBC-MSCs. MPTP was administered at a dose of 25 mg/kg for seven consecutive days. The motor dysfunction of the PD mouse model was validated using the rotarod and open field tests.

### Stereotaxic surgery

hNTSCs and hBM-MSCs were incubated for 5 min with trypsin–EDTA (Thermo Fisher Scientific), centrifuged, and then, the supernatant was removed. Mice were anesthetized, and their heads were fixed in a stereotaxic apparatus. After cleaning the surface of the skull, according to the Allen Brain Atlas, the distance between the two points of the lambda and bregma was identified. The hNTSCs and hBM-MSCs [1 × 10^5^ cells/10 µl N-Acetyl-L-cysteine(NAC)] were injected into the substantia nigra par compacta. The coordinates were as follows: AP = − 3.2, ML = 0.71 mm and DV = 4.6 mm.

### Behavior test

One week after MPTP IP injection, the motor function of the mice was validated by rotarod and open field tests, using a rotarod machine with falling sensors (MED-Associates Inc., FL, USA). In the rotarod test, ten mice for each group were placed side by side in a rotarod machine. The mice were allowed to remain in the machine for 5 min. Each session was performed after habituation. The acceleration of the rotation and rotation speed were set to 0–15 m/min and 0–50 rpm, respectively. Each rail on the machine was turned off as soon as the mouse fell off the platform. The rotarod test was conducted consecutively for four days. The open field test was conducted using SMART software (version 3.0) and an open field box (Panlab, MA, USA). The open field box was a 42 × 42 × 42 cm polyvinyl chloride box, which was monitored and recorded using a camera connected to the SMART 3.0 software. The camera measured the movements of the mice in the peripheral and central zones. Trajectory tracing, total travel distance, and time spent in the zone were measured to analyze the motor function of the animal.

### Flow cytometry

hNTSCs (1 × 10^5^ cells) were collected in a round bottom tube and resuspended to 100 μL of 5% FBS (Thermo Fisher Scientific) diluted in Dulbecco’s phosphate-buffered saline (DPBS; Thermo Fisher Scientific). Cells were stained with human CD90-PE conjugated (BD Bioscience, NJ, USA) and CD34 (FITC) at RT for 2 h. The cells were then evaluated using a FACSAria III (BD Biosciences) machine.

### Immunofluorescence staining

DA-Like cells were washed three times with DPBS (Thermo Fisher Scientific) and then fixed in cold methanol on ice for 15 min. After fixation, normal goat serum was used as a blocking buffer, for an hour at RT. Primary antibodies were diluted to 1:100 in blocking buffer and added to the cells. The cells were incubated overnight at 4 °C and then washed with DPBS three times. Secondary antibodies were diluted 1:500 in DPBS and incubated in the buffer for an hour. DAPI (Vector Laboratories) was diluted 1:5000 and incubated with the samples for 5 min at RT. The brain of MPTP-induced PD mouse model was fixed in 4% paraformaldehyde for 2 h and incubated in 30% of sucrose until it is crysectioned. The brain was sectioned 7 μm from the bregma to the end. The brain section slide was selected by referring to mouse brain atlas map and the slide was blocked with blocking buffer for an hour at RT. Primary antibody was diluted to 1:100 in blocking buffer and added to the slide. The slide was incubated overnight at 4 °C and washed with DPBS three times. Secondary antibody was diluted 1:500 in DPBS and incubated with the slide for an hour. DAPI was diluted 1:5000 and incubated for 5 min at RT. The fluorescence intensity of the cells and the slide were measured using a Zeiss LSM 800 Confocal Laser Scanning Microscope (Zeiss, Jena, Germany).

### Protein isolation

Brain proteins were extracted using RIPA buffer (LPS Solution, Daejeon, Korea) with EDTA-free Complete Ultra tablets and an EASYpack Protease Inhibitor Cocktail (Roche, Basel, Switzerland). The tissues were homogenized using a tissue grinder with lysis buffer containing proteinase and phosphatase inhibitors. Lysates were then sonicated for 10 secs in an ice bath sonication machine, followed by centrifuging at 14,000 × g for 20 min at 4 °C. The protein concentrations were measured using a bicinchoninic acid protein assay kit (Thermo Fisher Scientific).

### Immunoblotting

The proteins (15 μg) used for immunoblotting were mixed with 5 μL of NuPAGE™ LDS Sample Buffer (4x; Thermo Fisher Scientific), 2 μL of dithiothreitol (10x; Sigma-Aldrich, MO, USA), and distilled water, added to a final volume of 20 μL. The protein mixture was heated up to 75 °C for 10 min to denature the proteins. The protein mixture then underwent electrophoresis in a 10% SDS-PAGE gel for 40 min, in Invitrogen™ NuPAGE™ MOPS SDS Running Buffer (20x; Invitrogen). Proteins were separated by SDS-PAGE using the Trans-Blot Turbo Transfer System (Bio-Rad, Hercules, CA, USA). The PVDF membranes were blocked with 5% skim milk for 2 h at RT. The membranes were then washed three times with 0.1% tween-20 diluted in tris-buffered saline (0.1% T-TBS) for 5 min, three times, at RT. The transferred PVDF was then incubated with the primary antibody, either eukaryotic translation initiation factor 2 alpha subunit (EIF2α; Cell signaling technology, MA, USA), phosphorylated EIF2α activating transcription factor 4 (ATF4, Cell signaling technology), NEDD4L (Cell signaling), Yes-associated protein/transcription activator with PDZ binding motif (YAP/TAZ; Cell signaling), phosphorylated YAP/TAZ (Cell signaling), Large neutral amino acids transporter small subunit 1(LAT1; Cell signaling), or phosphorylated LAT1(Cell signaling). All the antibodies were diluted to 1:1000 with 0.1% sodium azide (Sigma-Aldrich) and incubated with the membranes at 4 °C overnight. The PVDF membranes were then washed with T-TBS for 10 min, three times, and then incubated with a goat anti-rabbit IgG antibody labeled with peroxidase (Vector Laboratory, Inc., CA, USA), diluted 1:5000, for 10 min at RT. The PVDF membranes were then analyzed using an LAS-3000 (FUJI PHOTO FILM CO., LTD, Tokyo, Japan).

### RNA isolation and cDNA synthesis

Midbrain tissue was homogenized using a tissue grinder and 300 μL of TRIzol^®^ reagent (Invitrogen). Tissue lysates were added to 60 μL of chloroform (DAEJUNG CHEMICALS & METALS Co., Ltd, Gyeonggi-do, Korea) and vortexed for 3 secs, to mix. The lysates were then centrifuged at 12,000 × g for 15 min at 4 °C. Supernatants were transferred to new 1.7 mL tubes, incubated in 0.5 mL of isopropanol (Sigma-Aldrich), and centrifuged at 12,000 × g for 15 min at 4 °C. The resulting RNA pellet was washed with 70% cold ethanol and centrifuged at 12,000 × g for 10 min at 4 °C. The RNA pellet was then dissolved in 30–50 μL of diethyl pyrocarbonate-treated water, according to the size of the pellet. The RNA was then incubated for 10 min at 65 °C. cDNA was synthesized from 1 µg of total RNA using the iScript™ cDNA Synthesis Kit (Bio-RAD).

### Poly-chain reaction (PCR) assay

The mRNA was isolated from the midbrain of each group using a manual protocol. cDNA was synthesized using the iScript™ cDNA Synthesis Kit (Bio-RAD). Then, 1 µg of cDNA was used for gene expression level analysis. CFX386 touch (Bio-RAD) was used to conduct qRT-PCR. The reaction efficiency and number of cycles were determined using innate software.

### Statistics

The Kruskal–Wallis and Mann–Whitney U post-hoc tests were used to compare the results across groups. SPSS version 22 (IBM Corporation, NY, USA) was used to determine statistically significant differences between groups. The results are expressed as mean ± standard deviation (SD). Differences were considered significant at *p < 0.05 versus Control; **p < 0.01 versus Control; ***p < 0.001 versus control; #*p* < 0.05 versus MPTP-saline and MPP^+^-saline; ##*p* < 0.01 versus MPTP-saline and MPP^+^-saline; ###*p* < 0.001 versus MPTP-saline and MPP^+^-saline.

## Results

### hNTSCs generation and characteristics analysis

**Fig. 1:**
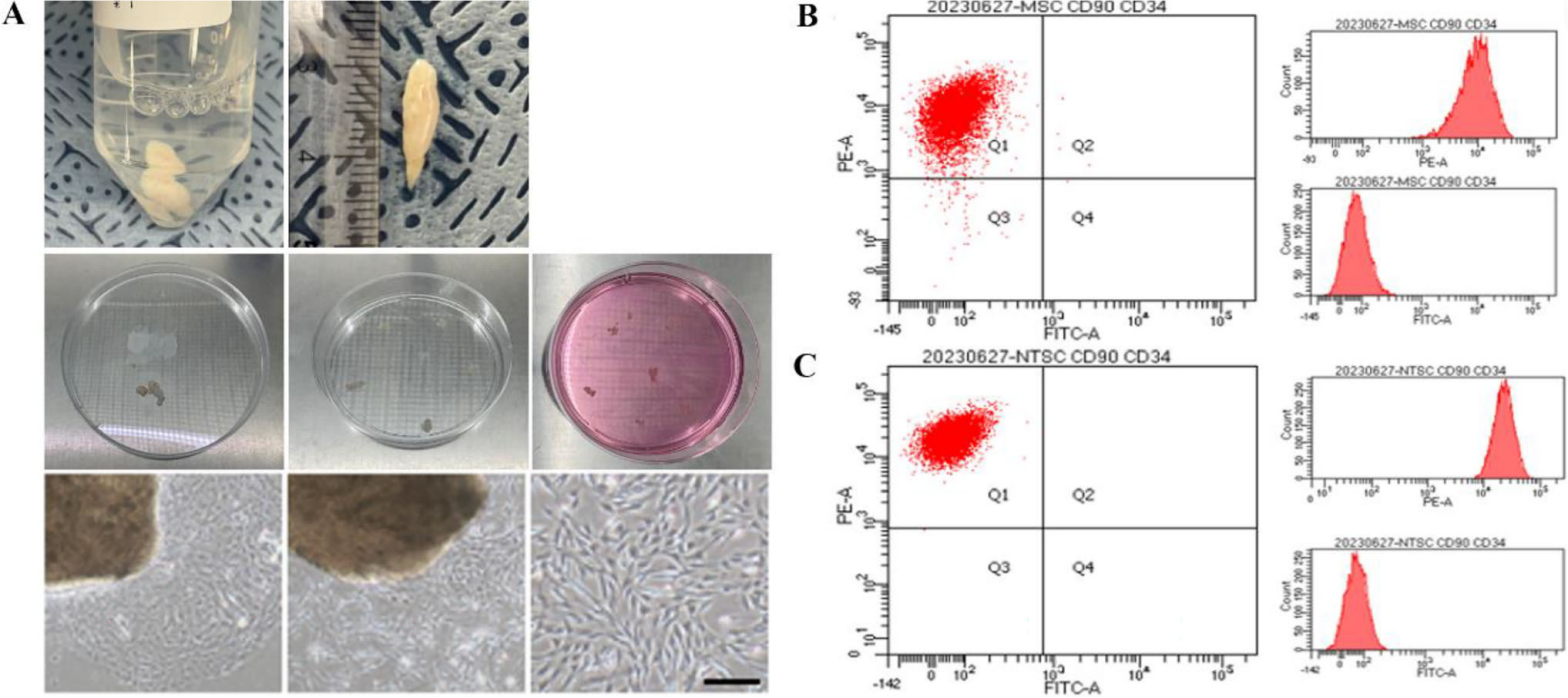
hNTSCs generated from nasal turbinate tissue. Nasal turbinate normal tissue donated from patients was chopped into 1 mm size and placed in a culture dish under the autoclaved cover slide. The dishes were then filled with culture media, and hNTSCs were grown from the tissue (**A**). Images show CD 90 (PE), CD 34 (FITC), stem cell marker flow cytometry data of hBM-MSCs (**B**). Images show the CD 90 (PE), CD 34 (FITC), and stem cell marker flow cytometry data of hNTSCs (**C**). bar = 50 μm

Nasal turbinate-derived normal tissues were obtained from each patient. The tissues were washed several times with DPBS (−/−) and reduced to a size of 1.5 cm. The tissue was chopped into a 1 mm size sample, placed in a cell culture dish under an autoclaved cover glass, and incubated in growth media for three weeks (Fig. 1A). hNTSCs expressed neuronal stem cell bio markers TUJ1, Nestin, P75, and NGFR [31]. To confirm the characteristics that are similar to those of hBM-MSCs, CD34 and CD90 that are considered stem cell surface markers. Notably, the hNTSCs showed similar characteristics to hBM-MSCs in the flow cytometry analysis (Fig. 1B, C).

### hNTSCs foster an MPP^+^-induced dopaminergic neuron-like cell niche

**Fig. 2:**
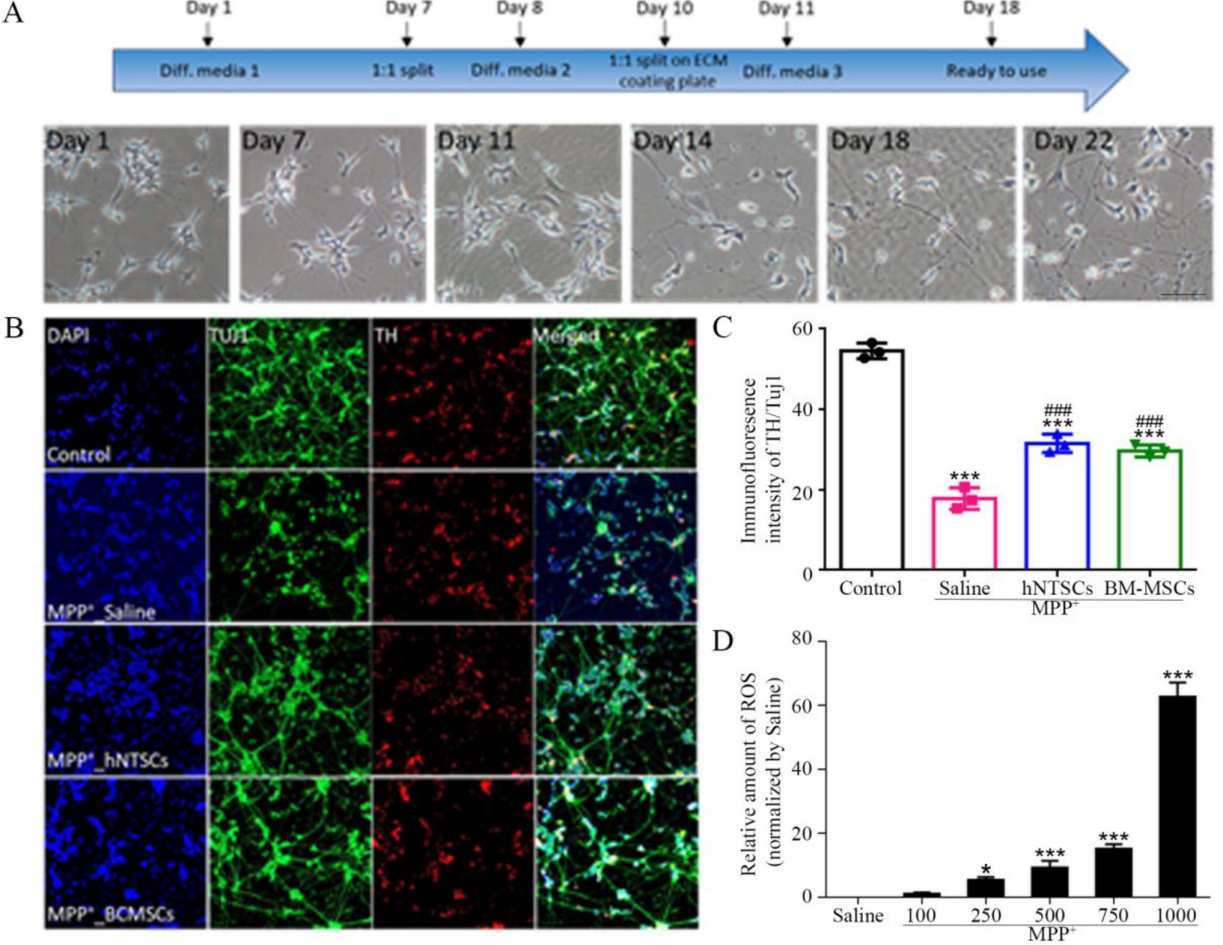
Differentiating SH-SY5Y cells to dopaminergic neuron-like cells. The diagram shows the morphological changes of SH-SY5Y cells during differentiation (**A**). The images show the TH and Tuj1 immunofluorescence staining of DA-Like cells after MPP^+^ treatment (**B**). The graph indicates the intensity of TH/Tuj1 of DA-Like cells (**C**). The graph indicates the impact of a ROS concentration gradient on SH-SY5Y derived DA-Like cells (**D**). *p < 0.05 versus control; ***p < 0.001 versus control; ###*p* < 0.001 versus MPP^+^-saline. bar = 50 μm

The SH-SY5Y human neuroblastoma cell line relies on gradual serum deprivation and retinoic acid supplementation, which reduce the period of time for differentiation into DA-Like cells. SH-SY5Y cells were cultured to differentiate into DA-Like cells in the bottom chamber of a trans-well chamber for three weeks (Fig. 2A). Three types of differentiation media composed of differentiation-related small molecules and chemicals were used (Table 1). The cells were completely differentiated by day 18 (Supplementary 1, 2). The fully differentiated DA-like cells were treated with a solution of 500 mM of MPP^+^ overnight. hNTSCs and hBM-MSCs were cultured in the upper chamber of a transwell culture dish. The relative TH/TUJ1 immunofluorescent staining intensity of DA-Like cells showed that cells treated with MPP^+^ saline had a significantly decreased intensity compared to that of the control group, MPP^+^_hNTSCs, and MPP^+^_hBM_MSCs. MPP^+^ hNTSCs and MPP^+^ hBM_MSCs showed increased relative intensity compared to MPP^+^ saline-treated cells. Notably, MPP^+^ hNTSCs showed no significant differences from MPP^+^ hBM_MSCs.

**Table 1:** Stem cell membrane bounded protein antibody (CD90, CD34) for flow cytometry analysis

Antibody	Company	Catalog number	Dilution Rate
Human CD90/Thy1 PE-conjugated Antibody	R&D Systems	FAB2067P	1:500
Human CD34 Alexa Fluor^®^ 488-conjugated Antibody	R&D Systems	FAB7227G	1:500
Anti-beta III Tubulin antibody—Neuronal Marker	Abcam	ab18207	1:500
Rabbit Tyrosine Hydroxylase antibody	Pel-Freez^®^ Biochemical	P40101-150	1:500
DAPI	Sigma Aldrich	D9564	1:5000

Dopaminergic neuron cell marker antibodies (beta III Tubulin; Tuj1, Tyrosine Hydroxylase; TH) for immunofluorescence staining

### hNTSCs modulate hippo-pathway signaling factors of MPP^+^ induced dopaminergic neuron-like cells

**Fig. 3:**
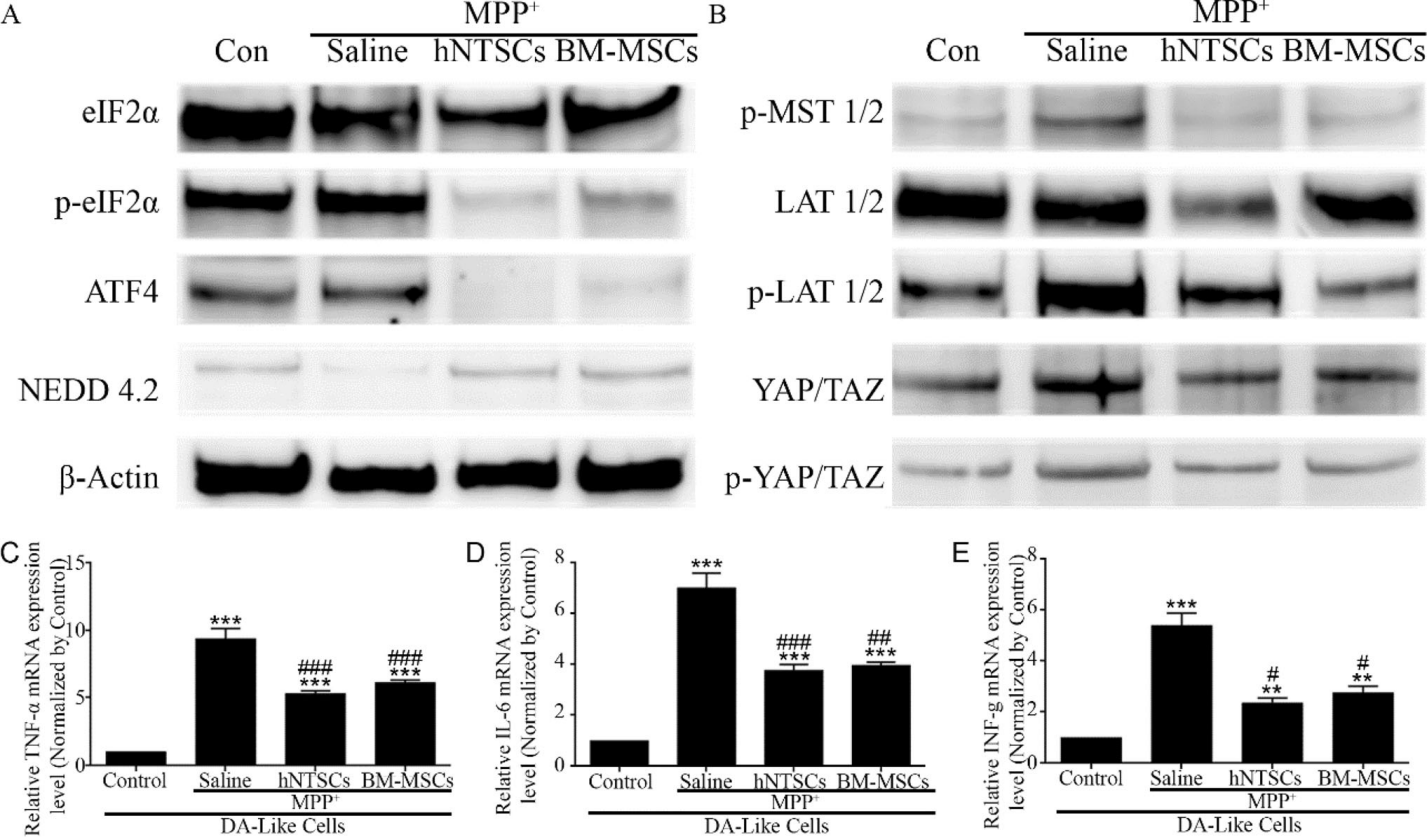
Images show the western blot results of ER-Stress factors, EIF2α, p-EIF2α, ATF4, NEDD4.2, and β-actin. The immunoblotting was performed on MPP^+^-treated DA-like cells (**A**). Images show the results for hippo-signaling pathway factors, p-MST1/2, LAT1/2, p-LAT1/2, YAP/TAZ, p-YAP/TAZ, upon immunoblotting of MPP^+^-treated DA-like cells (**B**). The graph indicates the relative gene expression levels of TNF-α, IL-6, and interferon-γ in DA-like cells (**C**–**E**). **p < 0.01 versus control; ***p < 0.001 versus control; #*p* < 0.05 versus MPP^+^-saline; ##*p* < 0.01 versus MPP^+^-saline; ###*p* < 0.001 versus MPP^+^-saline

MPP^+^-induced PD DA-like cells are damaged by ROS-mediated ER stress in the cytoplasm, and the Hippo signaling pathway is involved in cell death. The protein expression levels of phosphorylated EIF2α and ATF4 of the MPP^+^ saline group were significantly higher than those of control, MPP^+^_hNTSCs, and MPP^+^_hBM_MSCs cohorts. In contrast, the protein expression level of NEDD4.2, which inhibits the phosphorylation of LAT1, was lower than that of the control, MPP^+^_hNTSCs, and MPP^+^_hBM_MSCs groups (Fig. 3A). Furthermore, the protein expression levels of phosphorylated LAT1 and YAP/TAZ in the MPP^+^_saline group were significantly higher than those in the control, MPP^+^_hNTSCs, and MPP^+^_hBM_MSCs groups (Fig. 3B). The relative gene expression levels of inflammatory factors, TNF-α, IL-6, and INF-γ, were significantly decreased in MPP^+^_hNTSCs and MPP^+^_hBM-MSCs groups, compared to the MPP^+^_saline group (Fig. 3C–E). The sequence is informed in Table 2.

**Table 2:** Human origin primers information in this study

Gene	Direction	Sequence
TNF-α	Forward	5’-CTCTCTCTAATCAGCCCTCTGG-3’
TNF-α	Reverse	5’-GTTTGCTACAACATGGGCTACA-3’
IL-6	Forward	5’-AGAAAACAACCTGAACCTTCCA-3’
IL-6	Reverse	5’-ATGATTTTCACCAGGCAAGTCT-3’
Inf-γ	Forward	5’-TTGGGTTCTCTTGGCTGTTACT-3’
Inf-γ	Reverse	5’-ATCCGCTACATCTGAATGACCT-3’
β-actin	Forward	5’-GGGACCTGACTGACTACCTCAT-3’
β-actin	Reverse	5’-CCTTAATGTCACGCACGATTT-3’

Tumor necrosis factor alpha; TNF-α, Interleukin-6; IL-6, Interferon gamma; Inf-γ, actin beta; β-actin

### MPTP-induced Parkinson’s disease mouse model generation and validation

**Fig. 4:**
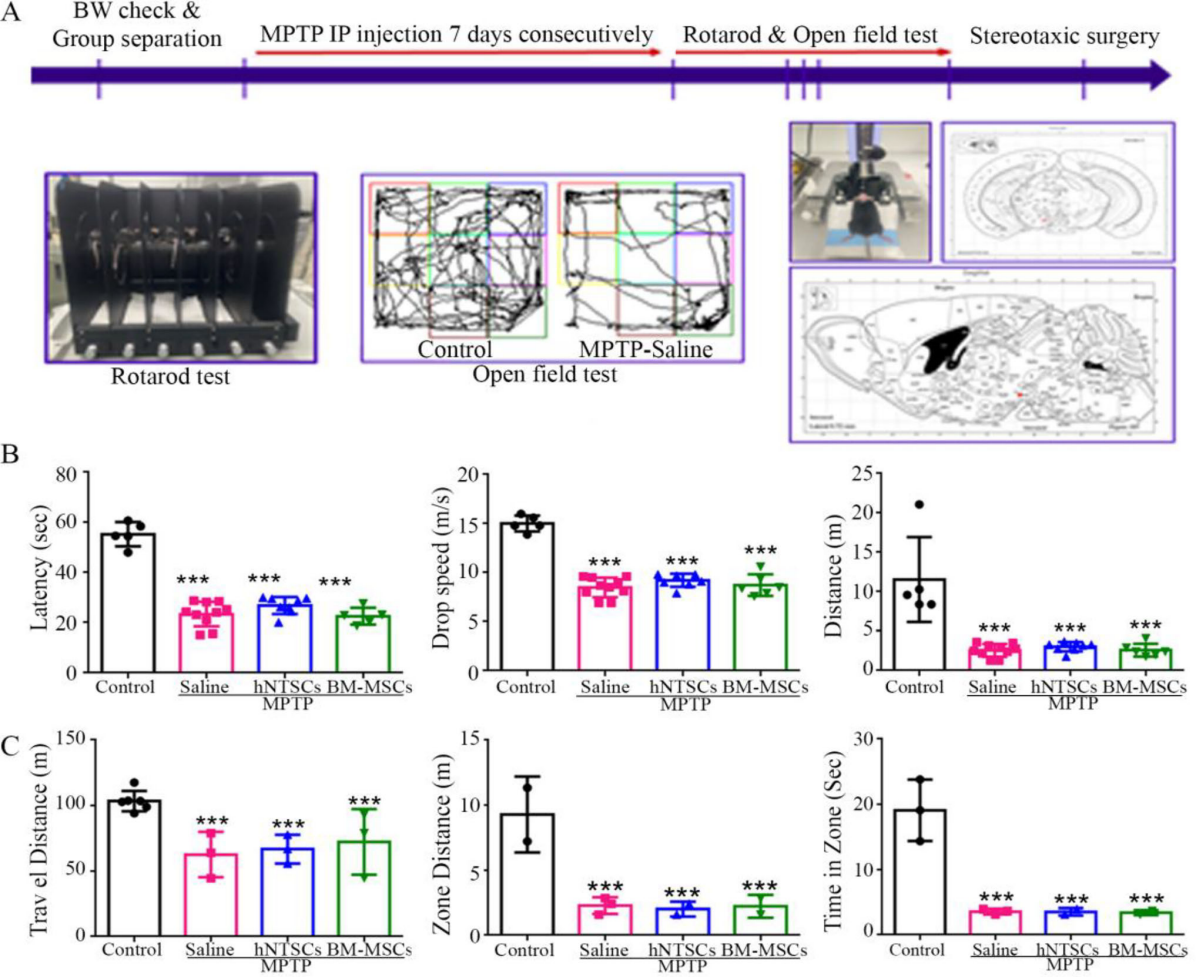
MPTP-mediated PD animal model generation and validation. The diagram indicates the MPTP-induced PD animal preparation schematic and a list of behavior tests (**A**). The graph shows the results of the first week of the Rotarod test in the MPTP-induced PD animal model (**B**). The graph shows the first week of the open field test of the MPTP-induced PD animal model (**C**). ***p < 0.001 versus control

Seven-week old male mice were used as a PD mimic animal model, and each group consisted of 10 mice. The mice were separated randomly and intraperitoneally injected with MPTP (25 mg/kg IP for a week, consecutively). The animals were validated by PD modeling using the rotarod and open field tests. Most mice injected with MPTP showed motor dysfunction, which was validated by the rotarod and open field tests. Latency, drop speed, and distance were significantly decreased in MPTP-injected mice (Fig. 4B). Furthermore, in the open field test, the MPTP-injected mice showed a significant decrease in motor function (Fig. 4C). The PD mouse model was then subjected to stereotaxic surgery two days after the behavioral test. The hNTSCs and hBM-MSCs were transplanted into the substantia nigra pars compacta via stereotaxic surgery (Fig. 4A).

### hNTSCs regulate hippo-pathway signaling factors of the MPTP-induced PD mouse model

**Fig. 5:**
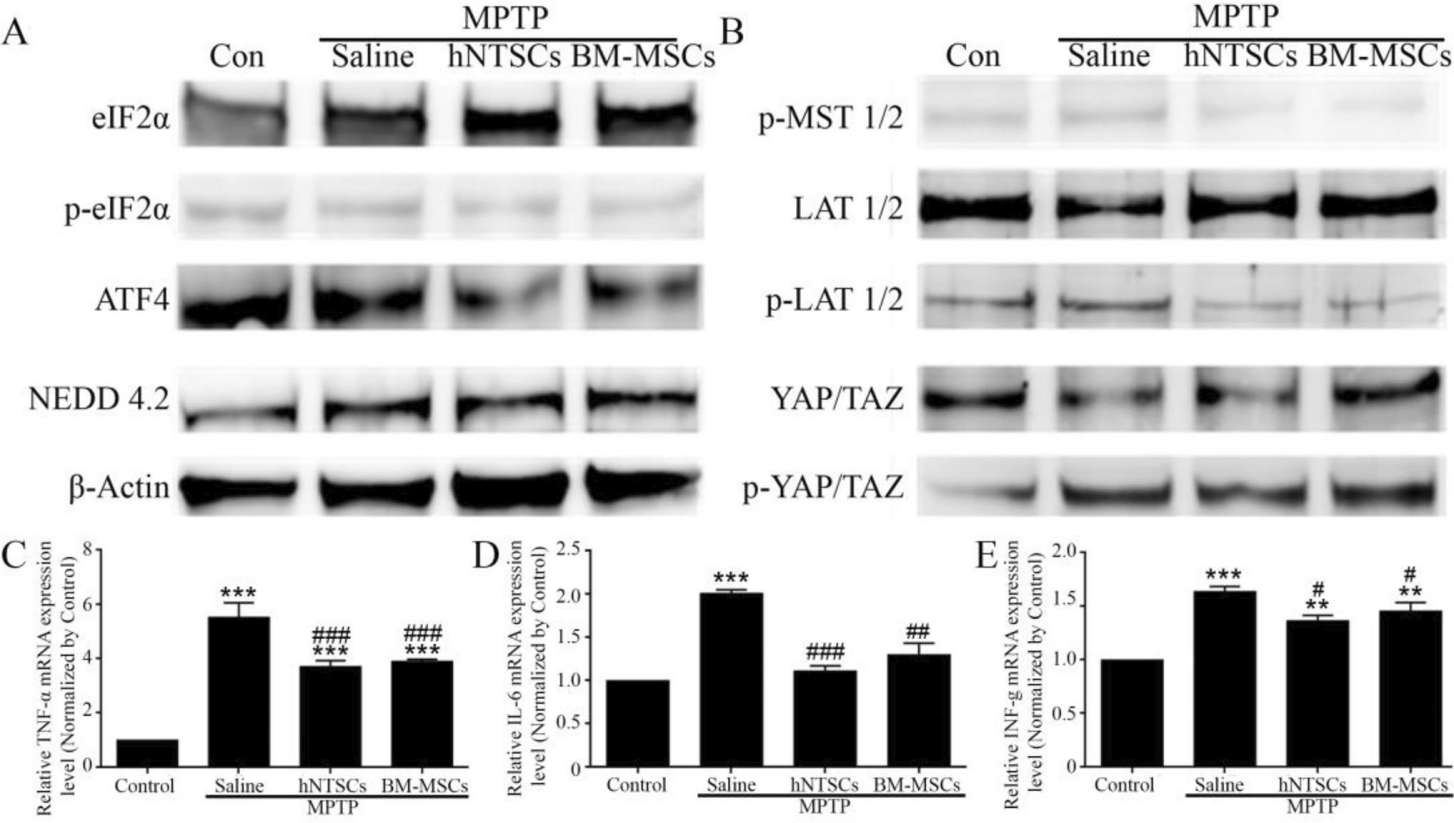
The images show the immunoblotting results for ER-Stress factors, EIF2α, p-EIF2α, ATF4, NEDD4.2, and β-actin from midbrain samples of MPTP-induced Parkinson’s model mice (**A**). Images indicate the immunoblotting results of hippo-signaling pathway factors, p-MST1/2, LAT1/2, p-LAT1/2, YAP/TAZ, p-YAP/TAZ, from mid-brain samples of MPTP-induced Parkinson’s model mice (**B**). The graphs indicate TNF-α, IL-6 and INF-γ gene expression levels (**C**–**E**). **p < 0.01 versus control; ***p < 0.001 versus control; #*p* < 0.05 versus MPTP-saline; ##*p* < 0.01 versus MPTP-saline; ###*p* < 0.001 versus MPTP-saline

The mice were sacrificed four weeks after cell transplantation. The midbrains were isolated and homogenized for immunoblotting and PCR analyses. eIF2α, p-eIF2α, ATF4, and NEDD4.2 primary antibodies were used to confirm the presence of ROS-mediated ER-stress. The protein expression level of p-eIF2α and ATF4 were downregulated in MPTP-hNTSCs and MPTP-hBM-MSCs, compared to those of the MPTP-saline group, and NEDD4.2 levels were upregulated (Fig. 5A). The protein levels of p-MST 1/2, p-LAT 1/2, and p-YAP were downregulated in MPTP-hNTSCs and MPTP-hBM-MSCs, compared to the respective levels in the MPTP-saline group (Fig. 5B). The inflammatory gene expression levels of TNF-α, IL-6, and INF-γ were significantly downregulated as well (Fig. 5C–E and Table 3).

**Table 3:** Mouse origin primers information in this study

Gene	Direction	Sequence
TNF-α	Forward	5’-CATGGATCTCAAAGACAACCAA-3’
TNF-α	Reverse	5’-CCTTGAAGAGAACCTGGGAGTA-3’
IL-6	Forward	5’-CTGCAAGAGACTTCCATCCAGT-3’
IL-6	Reverse	5’-TCCTCTGTGAAGTCTCCTCTCC-3’
Inf-γ	Forward	5’-CAAGTGGCATAGATGTGGAAGA-3’
Inf-γ	Reverse	5’-GGATTTTCATGTCACCATCCTT-3’
β-actin	Forward	5’-CCGTAAAGACCTCTATGCCAAC-3’
β-actin	Reverse	5’-GCAGTAATCTCCTTCTGCATCC-3’

Tumor necrosis factor alpha; TNF-α, Interleukin-6; IL-6, Interferon gamma; Inf-γ, actin beta; β-actin

### hNTSCs restore the dopaminergic neuron of MPTP-induced PD mouse model

The brains of the MPTP-induced PD mouse model were cut 7 μm cryo-section and stained to TH antibody to validate the influence of hNTSCs on dopaminergic neuron survival (Fig. 5A). The MPTP-hNTSCs and MPTP-hBM-MSCs group showed higher number of TH positive cells than those of MPTP-saline group statistically. However, MPTP-hNTSCs and MPTP-hBM-MSCs group showed less number of TH positive cells than those of control group. On the other hands, MPTP-hNTSCs group showed higher number of TH positive cells than those of MPTP-hBM-MSCs group statistically (Fig. 5B).

### The MPTP-induced PD mouse model presented recovered and improved motor function

**Fig. 6:**
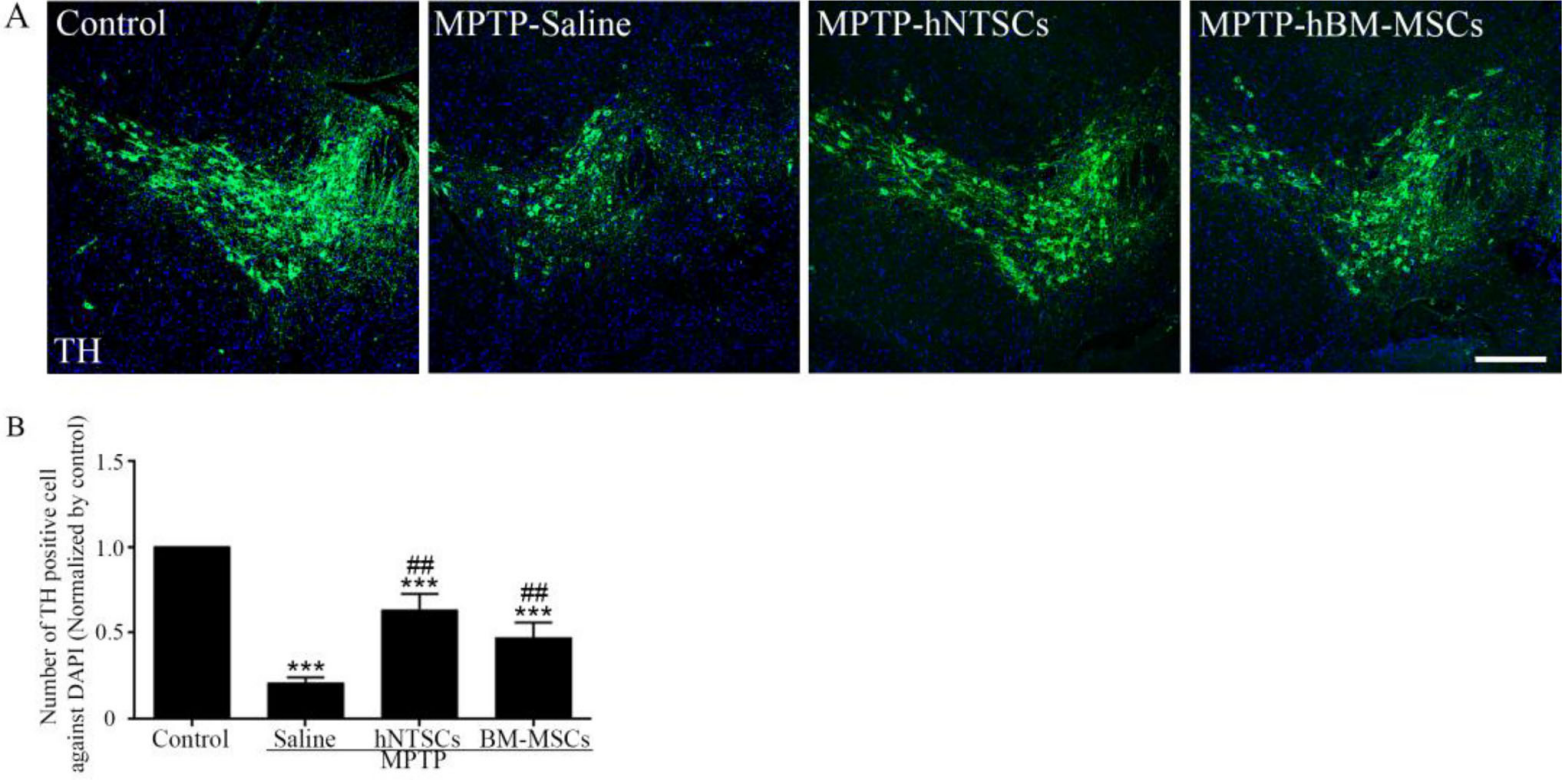
Presenting images indicate tyrosine hydroxylase (TH) positive immunofluorescence staining, DAPI (Blue), TH (Green) (**A**). The graph indicates the number of TH cell/DAPI (**B**). ***p < 0.001 versus control; ##*p* < 0.01 versus MPTP-saline. Scale bar = 200 μm

**Fig. 7:**
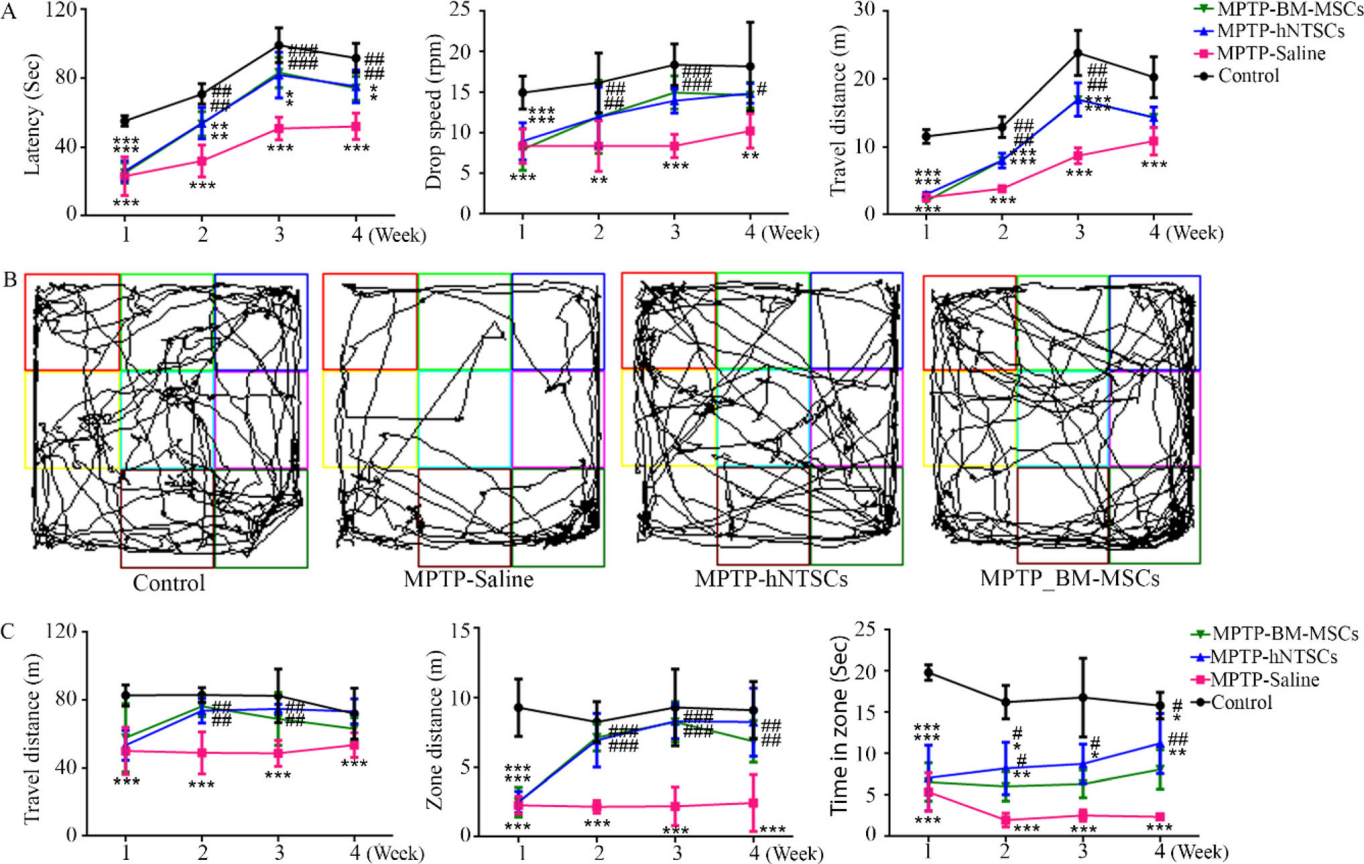
MPTP-induced PD animal model validation after cell transplantation. The graph shows the results of the Rotarod test over four weeks (**A**). The graph shows the result of the Rotarod test on the fourth week The images indicate the trajectory tracking of animal models in the open field test on the fourth week (**B**). The graph indicates the travel distance, zone distance and time in zone data over four weeks (**C**) *p < 0.05 versus control; **p < 0.01 versus control; ***p < 0.001 versus control; #*p* < 0.05 versus MPTP-Saline; ##*p* < 0.01 versus MPTP-Saline; ###*p* < 0.001 versus MPTP-Saline

The mice showed valid behavioral differences during the four weeks after cell transplantation. The rotarod test showed a gradual restoration of latency, drop speed, and travel distance of hNTSCs and hBM-MSCs, compared with those of the MPTP-saline group (Fig. 6A). In the open field trajectory test, the trace data also showed increased mobility of MPTP-hNTSCs and MPTP-hBM-MSCs, compared to that of the MPTP-saline group (Fig. 6B). In addition, the travel distance and time in the zone also reflected increased mobility (Figs. 6C and 7).

## Discussion

Stem cell therapy has been highlighted as a regenerative medicine for neurodegenerative diseases such as Alzheimer’s disease and Parkinson’s disease. Despite considerable efforts to elucidate the etiology of Parkinson’s disease, Parkinson’s disease modeling is still having difficulty implementing an accurate model. The human neuroblastoma SH-SY5Y cell line is an alternative source of human Parkinson’s disease modelling, and it is relatively easy to differentiate dopaminergic neuron-like cell phenotypes within three weeks upon lowering FBS to 1% and adding 10 μM retinoic acid. SH-SY5Y-derived dopaminergic neuron-like cells not only express DJ-1 protein and TH, which are extensively related to the early onset of PD, but also the dopamine transporter (DAT), which regulates dopamine homeostasis, incorporates MPP^+^, and can be utilized in MPP^+^-induced neurotoxicity [38, 39]. MPP^+^ induces ROS production, which disrupts the electron transportation system of the mitochondria and leads to DNA laddering. Also, alpha-synuclein (α-syn) protein aggregation commences the ROS stress in mitochondria and the α-syn overexpression and aggregation are affected by numeral genes such as apolipoprotein E (APOE). In Parkinson’s disease, APOE-ε4, one of the phenotypes of APOE, is well known to promote neurodegenerative disease. APOE-ε4 is highly expressed in PD patients and the overexpressed APOE-ε4 accelerates aggregating α-syn in the lesion, leading to ROS stress in the lesion [40]. In this study, we mimicked ros-mediated neurotoxicity by using the MPP^+^. In the transwell assay, MPP^+^-treated dopaminergic neuron-like cells showed shortened dendrites and neuronal cell death. In contrast, the Hippo signaling pathway is well known for its role in organ size, cancer development, and tissue regeneration. There are many hurdles to overcome in the perception of transplanting cells into lesions. First, it is important to improve the viability of the transplanted cells to regenerate their functions. The lesion of substantia nigra in Parkinson’s disease is upregulated and produced higher levels of inflammatory factors, TNF-α, Interleukin-6, and interferon gamma. The upregulation of these inflammatory factors and cytokines induces glial cell migration and exacerbates inflammation, which affects transplanted cell viability and leads to a lower engraftment rate. hNTSCs show characteristics similar to those of mesenchymal stem cells (MSCs), and the anti-inflammatory effects of MSCs are well known, though many studies before [27–29]. The trans-well *in vitro* assay showed that MPP^+^ saline-treated dopaminergic neuron-like cells upregulated inflammatory genes, TNF-α, and IL-6. However, after the hNTSCs were placed in the upper chamber, the expression of the inflammatory genes was downregulated, while anti-inflammatory gene expression levels were upregulated. The MPTP-induced *in vivo* test showed results similar to those of the *in vitro* test. Inflammatory gene expression levels in the mid-brain were downregulated in MPTP-hNTSCs and MPTP-hBC-MSCs groups, whereas anti-inflammatory gene expression levels were upregulated. In the live/dead assay with brain slides, the substantia nigra showed better cell viability in the lesions. Moreover, because hNTSCs are derived from the neural crest, they express neural lineage markers such as nestin, neural growth factor receptor p75, and sox2, which are more compatible with the central nervous system when engrafted. Although the same number of cells was transplanted into the MPTP-induced PD model, hNTSCs showed better cell viability than hBC-MSCs. Both stem cell types exhibit anti-inflammatory effects and relieve niches.

### Supplementary Information

Below is the link to the electronic supplementary material.

Supplementary file1 (DOCX 1217 kb)

## Data Availability

The datasets generated during and/or analysed during the current study are available from the corresponding author on reasonable request.
