# Supplementary material for: Human Nasal Inferior Turbinate-Derived Neural Stem Cells Improve the Niche of Substantia Nigra Par Compacta in a Parkinson’s Disease Model by Modulating Hippo Signaling
Source: Tissue Eng Regen Med. 2024 Apr 10;21(5):737–48. doi: 10.1007/s13770-024-00635-3 (PMC11187027; doi:10.1007/s13770-024-00635-3)
Supplement: Supplementary file 1 — Supplementary file1 (DOCX 1217 kb) [file 13770_2024_635_MOESM1_ESM.docx]

**Supplementary Figure 1**


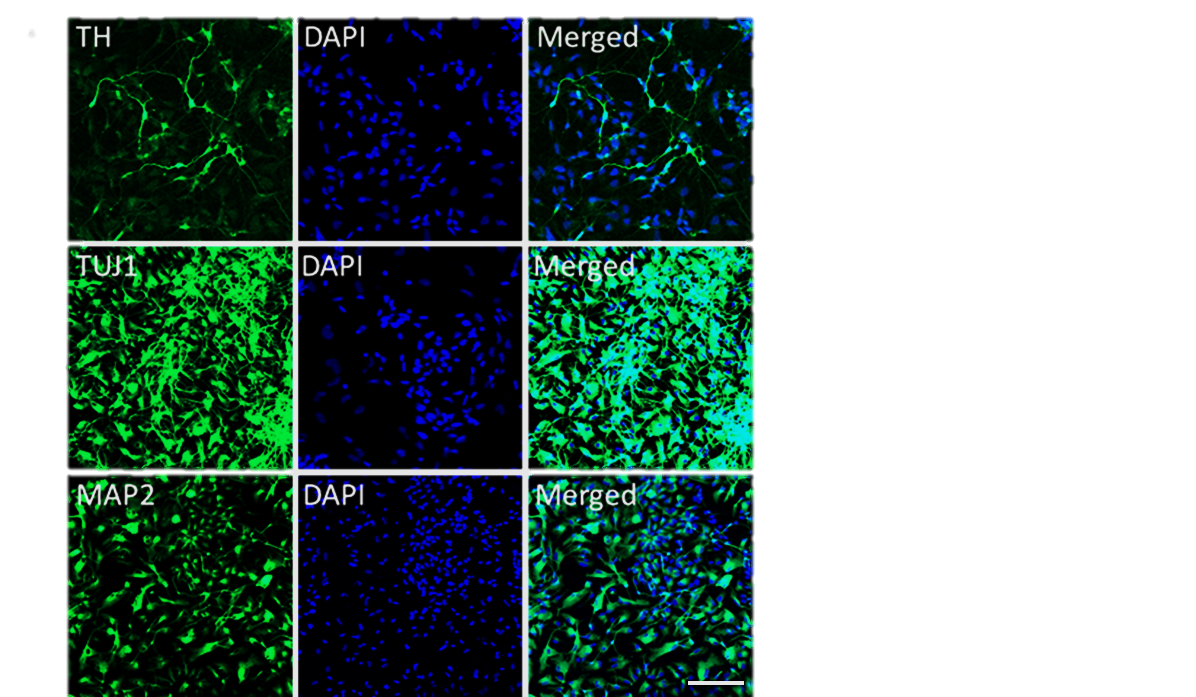


Supplementary Fig. 1. SHSY-5Y derived DA-Like Cells were completely differentiated by Day 18. Tyrosine hydroxylase(FITC), TUJ1(FITC) and MAP2(FITC) were immunofluorescence stained to validate differentiation of DA-Like Cells. DAPI (blue). Bar = 50 μm

**Supplementary Figure 2**


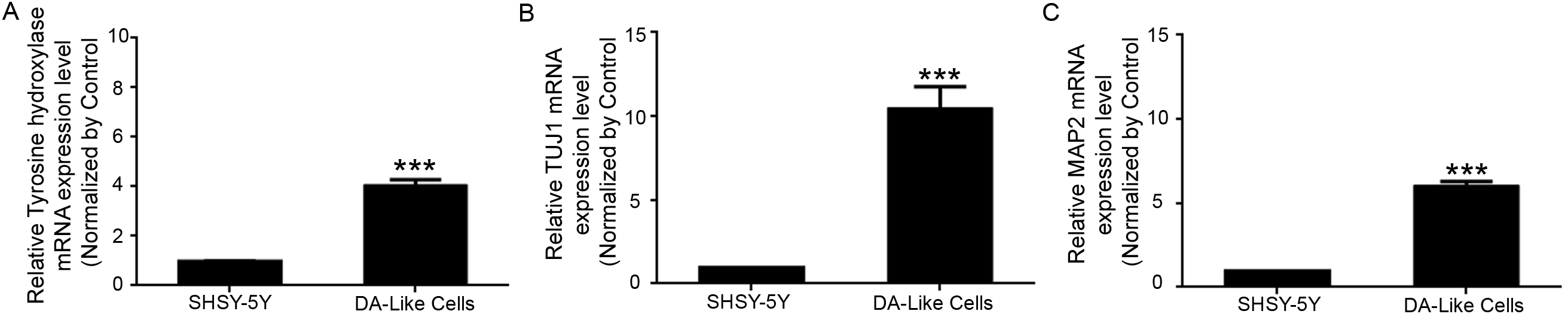


Supplementary Fig. 2. mRNA expression level of DA-Like Cells. The graphs indicate relative mRNA expression level of Tyrosine hydroxylase (A), TUJ1(B) and MAP2(C) in DA-Like Cells. *** p<0.001 vs. SH-SY5Y.
